# Prolonged versus intermittent β-lactam infusion in sepsis: a systematic review and meta-analysis of randomized controlled trials

**DOI:** 10.1186/s13613-024-01263-9

**Published:** 2024-02-18

**Authors:** Yang Zhao, Bin Zang, Qian Wang

**Affiliations:** 1 Department of Critical Care Medicine, Shengjing Hospital of China Medical University, 36 Sanhao Street, Shenyang, 110000 China; 2 Department of Emergency, The Fourth Affiliated Hospital of China Medical University, 4 Chongshan East Road, Shenyang, 110000 China

**Keywords:** Antibiotics, Cephalosporins, Carbapenems, Cephalosporins, Penicillin, Pharmacokinetics, Pharmacodynamics, Sepsis

## Abstract

**Background:**

The two latest studies on prolonged versus intermittent use of β-lactam antibiotics in patients with sepsis did not reach consistent conclusions, further contributing to the controversy surrounding the effectiveness of the prolonged β-lactam antibiotics infusion strategy. We conducted a systemic review and meta-analysis to evaluate the efficacy and safety of prolonged and intermittent β-lactam infusion in adult patients with sepsis.

**Methods:**

We systematically searched PubMed, EMBASE, and Cochrane Library databases for original randomized controlled trials comparing prolonged and intermittent β-lactam infusion in sepsis patients. A random-effects model was used to evaluate mortality, clinical success, microbiological success, and adverse events. We also conducted subgroup analyses to explore the impact of various factors on the mortality rates. Relative risk (RR) and corresponding 95% confidence intervals (CIs) were used to calculate the overall effect sizes for dichotomous outcomes. This meta-analysis was registered in PROSPERO (CRD42023463905).

**Results:**

We assessed 15 studies involving 2130 patients. In our comprehensive assessment, we found a significant reduction in all-cause mortality (RR, 0.83; 95% CI 0.72–0.97; P = 0.02) and a notable improvement in clinical success (RR, 1.16; 95% CI 1.03–1.31; P = 0.02) in the prolonged infusion group compared to the intermittent infusion group, whereas microbiological success did not yield statistically significant results (RR, 1.10; 95% CI 0.98–1.23; P = 0.11). No significant differences in adverse events were observed between the two groups (RR, 0.91; 95% CI 0.64–1.29; P = 0.60). Additionally, remarkable conclusions were drawn from subgroup analyses including studies with sample sizes exceeding 20 individuals per group (RR, 0.84; 95%CI 0.72–0.98; P = 0.03), research conducted post-2010 (RR, 0.84; 95%CI 0.72–0.98; P = 0.03), cases involving infections predominantly caused by Gram-negative bacteria (RR, 0.81; 95%CI 0.68–0.96; P = 0.02), as well as the administration of a loading dose (RR, 0.84; 95% CI 0.72–0.97; P = 0.02) and the use of penicillin (RR, 0.61; 95% CI 0.38–0.98; P = 0.04).

**Conclusions:**

Compared to intermittent infusion, prolonged infusion of β-lactam antibiotics significantly decreases all-cause mortality among patients with sepsis and enhances clinical success without increasing adverse events.

**Supplementary Information:**

The online version contains supplementary material available at 10.1186/s13613-024-01263-9.

## Introduction

Sepsis is a severe inflammatory syndrome caused by a dysregulated host response to infection [1]. It has been declared a top priority in global public health by the World Health Organization, contributing to approximately 20% of all-cause deaths worldwide [2]. Sepsis and septic shock represent a growing global burden owing to their increasing incidence [3, 4]. Antimicrobial treatment is the primary cornerstone in managing sepsis and septic shock [5].

β-Lactam antibiotics are the most widely used broad-spectrum antibiotics globally, especially for critically ill patients [6–8]. They exhibit time-dependent pharmacodynamics, wherein maintaining a drug concentration above the minimum inhibitory concentration (MIC) for an adequate duration is crucial for efficacy [7, 9]. β-Lactam antibiotics are traditionally administered intermittently [10]. However, pharmacokinetic research indicates that extending the infusion time can help maintain constant serum levels, potentially enhancing the duration above the MIC and, consequently, its effectiveness [11].

The Surviving Sepsis Campaign has weakly recommended the use of prolonged infusion of β-lactam antibiotics for adults with sepsis or septic shock as a maintenance approach (following an initial bolus) instead of the conventional bolus infusion [3]. However, research on the prolonged and intermittent use of β-lactam antibiotics in patients with sepsis has continued with two studies published in 2023 [12, 13]. These studies did not yield consistent conclusions, further contributing to the controversy surrounding the effectiveness of the prolonged β-lactam antibiotic infusion strategy. Several systematic reviews have attempted to assess the effectiveness of this method but failed to arrive at a consensus [9, 14]. It is noteworthy that the International consensus recommendations for the use of prolonged-infusion β-lactams published in August 2023 did not include these two 2023 randomized controlled trials (RCTs), underscoring the need for this additional systematic review and meta-analysis [15].

We aimed to perform a systematic review and meta-analysis by integrating RCTs on prolonged and intermittent infusion of β-lactam antibiotics among patients with sepsis. We evaluated data on all-cause mortality and clinical success and performed subgroup analysis.

## Materials and methods

This meta-analysis was performed following the Preferred Reporting Items for Systematic Reviews and Meta-Analysis (PRISMA) statement [16]. The protocol of our review was registered in PROSPERO (CRD42023463905) in September 2023. Ethical approval was not required for this systematic review and meta-analysis.

### Search strategy

We searched the electronic databases PubMed, EMBASE, and Cochrane Library for original RCTs evaluating the efficacy and safety of prolonged and intermittent β-lactam antibiotics infusion in adult patients with sepsis (search last updated October 2023). Our search terms included: ((sepsis) OR (septic*) OR (Systemic Inflammatory Response Syndrome)) AND ((discontinuous) OR (intermittent) OR (interval)) AND ((continuous) OR (extended) OR (prolonged)) AND ((administration) OR (infusion) OR (intravenous)) AND ((beta-lactam) OR (penicillin) OR (piperacillin) OR (cephalosporin) OR (meropenem) OR (imipenem) OR (doripenem) OR (ticarcillin) OR (cefepime) OR (ceftazidime) OR (cefoperazone) OR (monobactam) OR (aztreonam) OR (ertapenem) OR (cefazolin) OR (sulbactam) OR (tazobactam)). We limited our search to studies reported in the English language.

### Study selection

Two reviewers (QW and YZ) independently screened the abstracts. RCTs were included according to the following criteria: (1) studies including patients aged 18 years or older; (2) studies including patients admitted to the ICU due to sepsis or septic shock (definitions of sepsis not restricted to the latest sepsis-3 [1] definition and include sepsis-2 [17] definition); (3) studies with indication for β-lactam use; (4) studies employing either a prolonged β-lactam infusion strategy (24-h continuous or extended time, i.e., greater than 1 h but not continuous intravenous infusion) or an intermittent β-lactam infusion strategy (intravenous infusion lasting up to 1 h); and (5) studies reporting mortality outcomes and efficacy parameters, such as clinical and microbiological success. Studies were excluded if they included: (1) patients < 18 years old; (2) pregnant patients; (3) patients with acute or chronic renal failure and those who needed dialysis; (4) patients who received previous therapy with β-lactam for more than 24 h before randomization; (5) patients with immunodeficiency or patients taking immunosuppressants; (6) patients with neutropenia (absolute neutrophil count < 1000 cells/mm^3^); and (7) patients with hypersensitivity or allergy to β-lactam.

Two investigators (YZ and QW) independently conducted full-text reviews of eligible studies after excluding studies based on title and abstract. Additional research was conducted by searching the reference lists of the reviewed articles. Conflicts between the reviewers were resolved by a third reviewer (BZ). We used EndNote 20.0 for the screening process.

### Data extraction

Data extraction from the included studies was independently done by the two reviewers (YZ and QW). The following data were extracted: authors, year of publication, country, number of patients, mean age, gender, Acute Physiology and Chronic Health Evaluation II (APACHE II) and Sequential Organ Failure Assessment scores, pathogen involved, and specific β-lactam antibiotics used along with dosages and route of administration.

### Study endpoints

All-cause mortality at any timepoint was set as the primary outcome measure. Regarding the selection of different time points for mortality, our prioritization is as follows: hospital mortality, 28-day mortality, 90-day mortality, ICU mortality, and other mortality data. For studies reporting survival rates, we will incorporate these figures into the analysis after converting them into mortality rates. The secondary outcomes were clinical success, microbiological success, and adverse events. Clinical success was defined as the complete or partial resolution of temperature, clinical signs and symptoms of infection, and leukocytosis. Microbiological success was defined as the eradication or presumed eradication of microbiological etiology (eradication: cultures are negative and remain negative upon continued culture; presumed eradication: repeat cultures are not obtained owing to the absence of culture material in a patient who has responded to therapy).

### Subgroup analysis

We conducted a subgroup analysis to explore the impact of various factors on mortality, such as baseline age and APACHE II score, sample size, publication year, pathogen, use of loading dose in the prolonged infusion group, and β-lactam classes.

### Assessment of risk bias

The quality of the included studies was independently assessed by two reviewers (QW and YZ) based on the Cochrane Collaboration tool. The risk of bias was assessed in seven prespecified domains: random sequence generation, allocation concealment, blinding of participants and personnel, blinding of outcome assessment, incomplete outcome data, selective reporting, and other biases [18]. We performed a visual analysis of funnel plots and Egger’s test to assess publication bias [19, 20].

### Statistical analysis

Statistical analysis was conducted using STATA version 16.0 (Stata Corp., College Station, TX 77845, USA). We used relative risk (RR) with corresponding 95% confidence intervals (CIs) to calculate the overall effect sizes for dichotomous outcomes. To account for potential heterogeneity among the studies, we employed a random-effects model. Heterogeneity was assessed using the Higgins’ I^2^ statistic and Cochran’s Q test. We also conducted a sensitivity analysis to evaluate the robustness of our study outcomes.

## Results

### Study selection

**Fig. 1:**
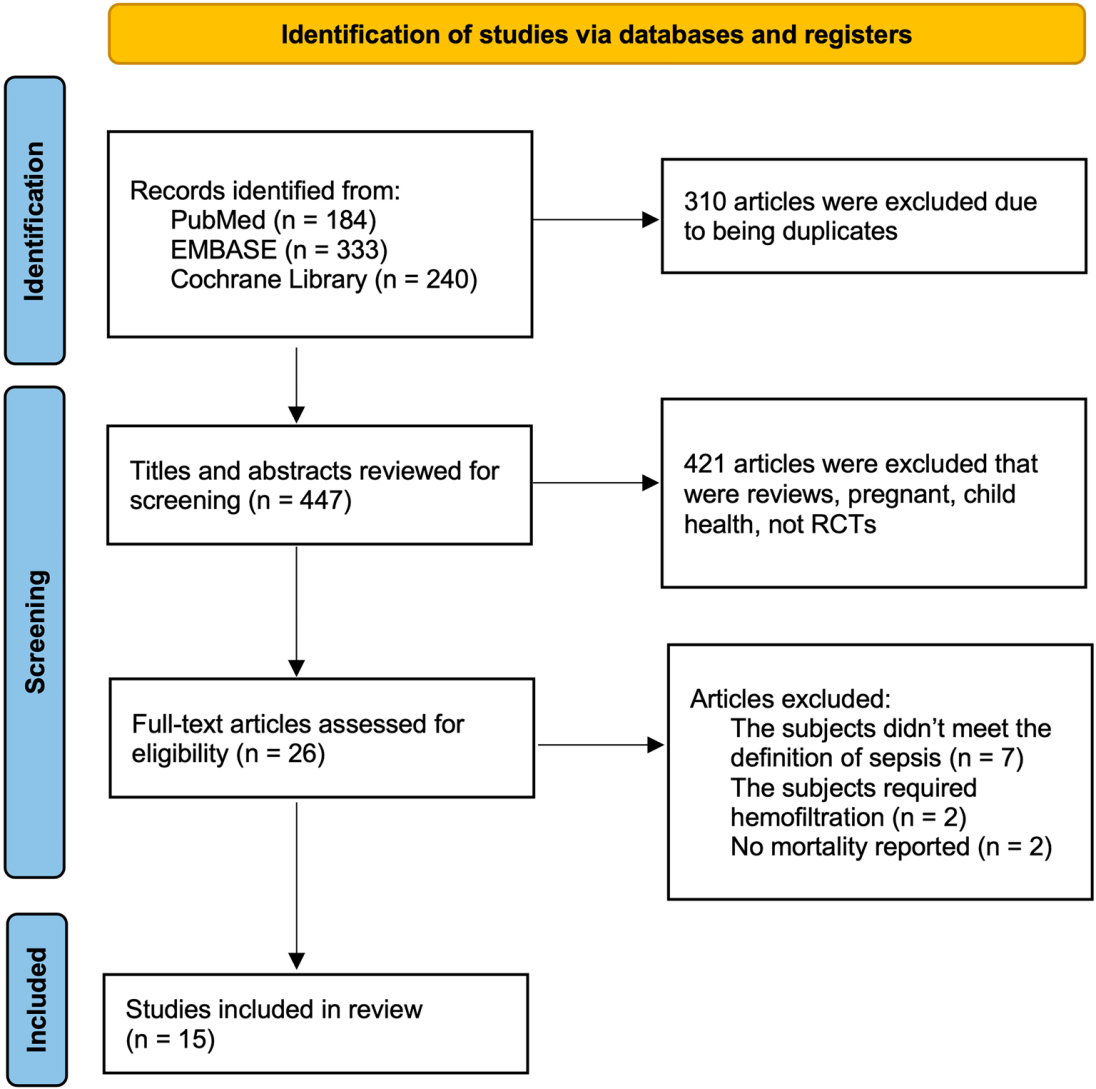
Flow diagram of the review

According to our search strategy, 757 articles were identified. Of these, 310 duplicate studies were excluded from the analysis. After reading the titles and abstracts, 421 articles were excluded because they did not meet the eligibility criteria. Out of the 26 remaining articles, 11 studies were excluded upon reading the full texts. Ultimately, 15 studies comprising 2,130 patients were included in this meta-analysis [12, 13, 21–33]. All 15 studies reported mortality outcomes in patients with sepsis who were treated with β-lactam antibiotics. The search process is illustrated in Fig. 1 using the PRISMA Flow Diagram [34].

### Study and patient characteristics

The characteristics of all included studies are presented in Table 1. Five RCTs were conducted in Asia [12, 22, 30–32], four in Oceania [23–26], three in multiple countries [13, 28, 29], two in Europe [27, 29], and one in North America [21]. Three RCTs used cephalosporin alone [23, 32, 33], four RCTs used carbapenems alone [13, 24, 27, 31], and five used penicillin alone [12, 21, 22, 25, 26]. The other RCTs involved multiple β-lactam antibiotics [28–30]. Apart from the four RCTs [21, 23, 28, 29] that involved treatment for mixed bacterial sepsis, most of the other RCTs predominantly dealt with Gram-negative bacteria. Two RCTs presented data on hospital mortality [12, 27], while one RCT reported 28 day mortality [13] and another focused on ICU mortality [31]. Additionally, for three RCTs [28–30], we converted 30-day/90-day survival rates and hospital survival rate into mortality rates. Eight RCTs simply provided data on deaths, mortality, or survival without further classifying the type of mortality [21–26, 32, 33]. 1060 patients received β-lactam therapy by prolonged infusion, while 1070 patients received β-lactam intermittent dosing. Among these patients, 1410 patients from 10 RCTs [12, 21, 23, 25, 27–33] were included in the clinical success group, 462 patients from 5 RCTs [21, 23, 27, 31, 33] were included in the microbiological success group, 1066 patients from 4 RCTs [12, 21, 27, 29] were included in the adverse event group. All studies were assessed for the risk of bias using the Cochrane Collaboration tool (Additional file 1: Figure S1 and Additional file 2: Figure S2).

**Table 1:** Characteristics of included studies

Author	Study design	Infection type	Country	Participants (N) PI vs II	Mean/median age (years) PI vs II	Female (N) PI vs II	Mean/median APACHE II score PI vs II	Pathogen	Antibiotics
Monti et al. [13]	Double-blind, RCT	Sepsis/septic shock	Croatia, Italy, Kazakhstan, and Russia	303 vs 304	66 vs 63	108 vs 95	44 vs 43^a^	Mostly Gram-negative	Meropenem
Mirjalili et al. [12]	Assessor-blind RCT	Sepsis/septic shock	Iran	68 vs 68	54 vs 53	31 vs 30	19.1 vs 19.2	Gram-negative	Ampicillin/sulbactam
Zhao et al. [31]	RCT	Sepsis/septic shock	China	25 vs 25	68 vs 67	15 vs 14	19.4 vs 19.7	Gram-negative	Meropenem
Abdul-Aziz et al. [30]	Open-label RCT	Severe sepsis	Malaysian	70 vs 70	54 vs 56	24 vs 20	21 vs 21	Mostly Gram- negative	Piperacillin/tazobactam, cefepime, meropenem
Dulhunty et al. [29]	Double-blind, RCT	Severe sepsis	Australia, New Zealand, Hong Kong	212 vs 220	64 vs 65	82 vs 85	21 vs 20	Mixed	Piperacillin/tazobactam, Ticarcillin/clavulanate, Meropenem
Dulhunty et al. [28]	Double-blind RCT	Severe sepsis	Australia and Hong Kong	30 vs 30	54 vs 60	7 vs 11	21 vs 23	Mixed	Piperacillin/tazobactam, Ticarcillin/clavulanate, Meropenem
Chytra et al. [27]	Open-label RCT	Sepsis	Plzen	106 vs 108	45 vs 47	42 vs 37	21.4 vs 22.1	Mostly Gram-negative	Meropenem
Roberts et al. [26]	Open-label RCT	Sepsis	Australia	8 vs 8	30 vs 41	2 vs 3	20 vs 24	Gram-negative	Piperacillin/tazobactam
Roberts et al. [24]	Open-label RCT	Sepsis	Australia	5 vs 5	57 vs 55	1 vs 2	NA	Gram-negative	Meropenem
Roberts et al. [25]	Open-label RCT	Sepsis	Australia	6 vs 7	25 vs 42	0 vs 3	17.5 vs 24.0	Gram-negative	Piperacillin/tazobactam
Roberts et al. [23]	Open-label RCT	Sepsis	Australia	29 vs 28	43 vs 52	13 vs 11	18.8 vs 16.4	Mixed	Ceftriaxone
Rafati et al. [22]	RCT	Sepsis	Tehran	20 vs 20	50 vs 48	8 vs 5	16.4 vs 14.2	Gram- negative	Piperacillin
Lau et al. [21]	Open-label RCT	Sepsis	the United States	128 vs 130	50 vs 49	47 vs 55	7 vs 7	Mixed	Piperacillin/tazobactam
Georges et al. [33]	Open-label RCT	Sepsis	France	26 vs 24	50 vs 46	5 vs 4	45 vs 44^a^	Mostly Gram-negative	Cefepime
Angus et al. [32]	RCT	Septicemic melioidosis	Thailand	10 vs 11	48 vs 43	1 vs 7	15 vs 21	Gram-negative	Ceftazidime

^a^Simplified Acute Physiology Score II (SAPS II); PI, prolonged infusion; II, intermittent infusion; NA, not available; APACHE II, Acute Physiology and Chronic Health Evaluation II; h, hour

### Outcome

**Fig. 2:**
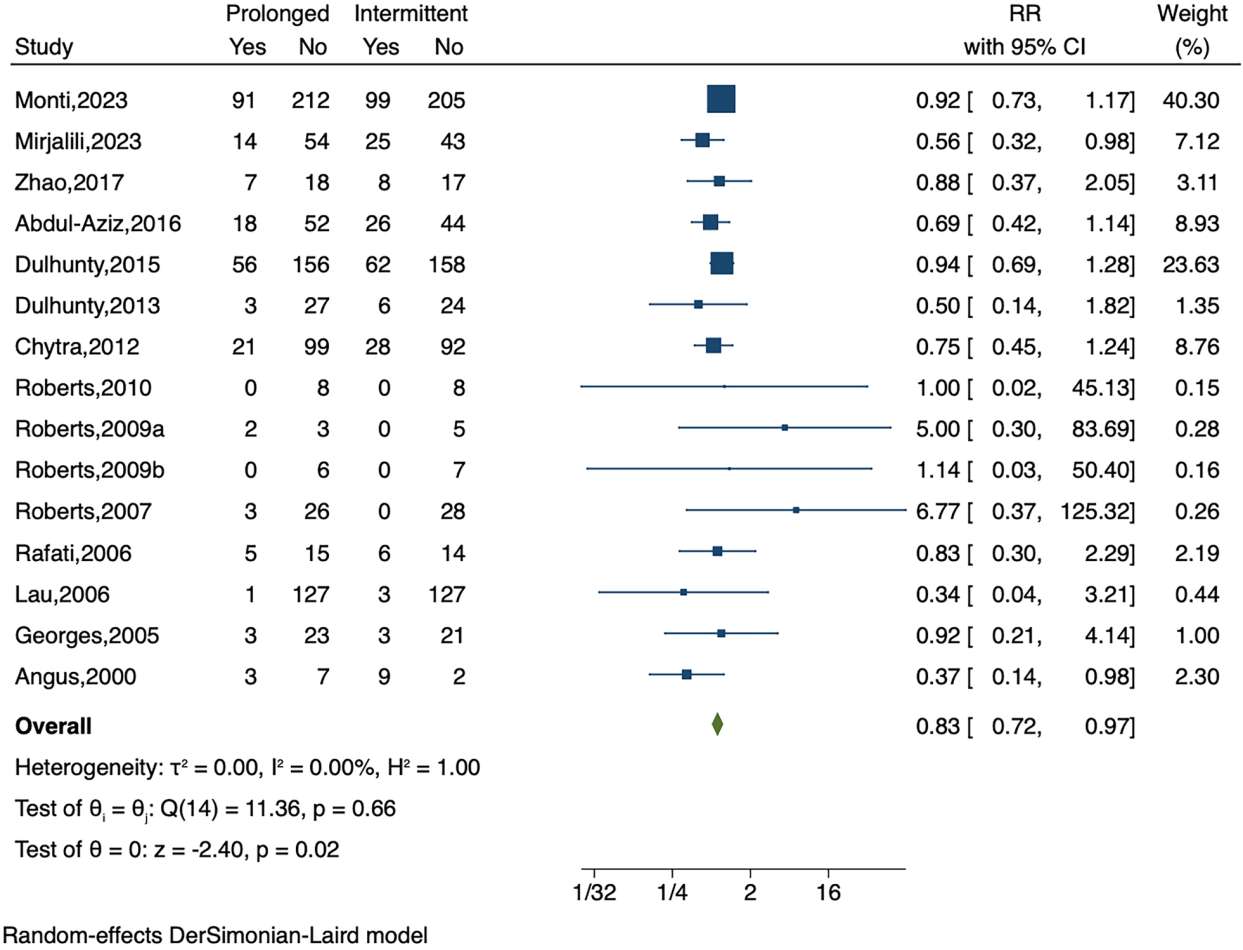
Forest plot of all-cause mortality. Prolonged versus intermittent infusion of β-lactam antibiotics among patients with sepsis. The points and the bars represent the relative risk (RR) and 95% confidence intervals (CIs). RR, relative risk; CI, confidence interval

**Fig. 3:**
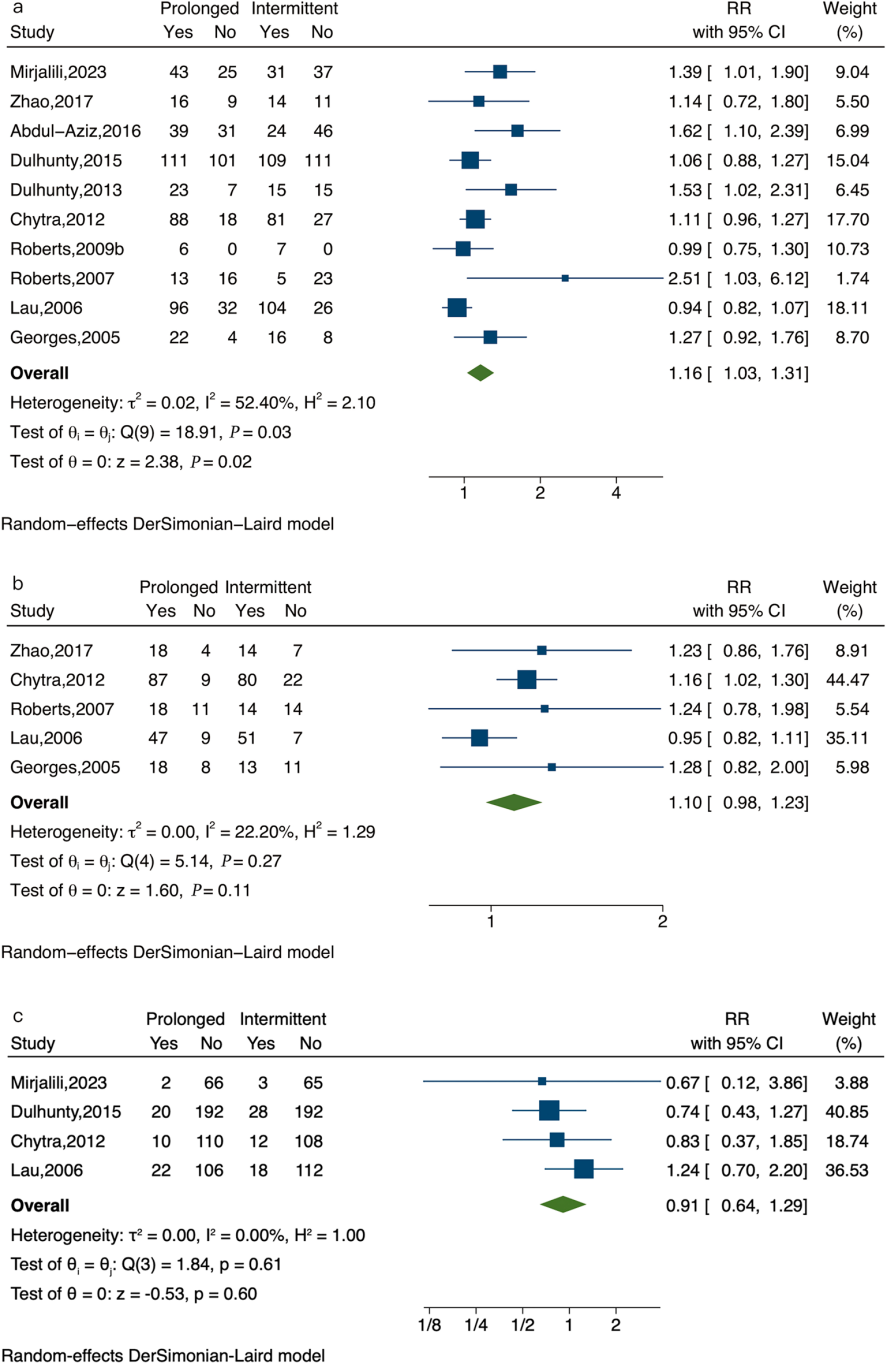
Forest plot of clinical success (**a**), microbiological success (**b**), adverse events (**c**). Prolonged versus intermittent infusion of β-lactam antibiotics among patients with sepsis. The points and the bars represent the relative risk (RR) and 95% confidence intervals (CIs). *RR* relative risk, *CI* confidence interval

The combined study population demonstrated a statistically significant reduction in all-cause mortality with prolonged infusion compared to intermittent infusion (Fig. 2; RR, 0.83; 95% CI 0.72–0.97; P = 0.02). Regarding the secondary outcomes, clinical success significantly improved in the prolonged infusion group compared to the intermittent infusion group (Fig. 3a, RR, 1.16; 95% CI 1.03–1.31; P = 0.02), while microbiological success did not yield statistically significant results (Fig. 3b; RR, 1.10; 95% CI 0.98–1.23; P = 0.11). There were no significant differences in the adverse events between the two groups (Fig. 3c; RR, 0.91; 95% CI 0.64–1.29; P = 0.60).

### Subgroup analysis on mortality

Mortality reduction was observed in subgroups receiving prolonged infusion, including studies with sample sizes exceeding 20 individuals per group (RR, 0.84; 95%CI 0.72–0.98; P = 0.03), research conducted post-2010 (RR, 0.84; 95%CI 0.72–0.98; P = 0.03), and cases involving infections predominantly caused by Gram-negative bacteria (RR, 0.81; 95%CI 0.68–0.96; P = 0.02). The use of a loading dose for prolonged β-lactam infusion resulted in a significant reduction in mortality (RR, 0.84; 95% CI 0.72–0.97; P = 0.02). Prolonged infusion demonstrated a tendency for reduced mortality compared to intermittent infusion, irrespective of APACHE II scores being above or below 20. This trend was consistent in populations both above and below 50 years of age. Except for three RCTs [28–30] that used combinations of β-lactam antibiotics, we conducted subgroup analyses on the twelve RCTs that used single β-lactams to assess mortality. Notably, the most pronounced efficacy of prolonged infusion was observed within the penicillin group (RR, 0.61; 95% CI 0.38–0.98; P = 0.04). (Table 2).

**Table 2:** Subgroup analyses on mortality

	Studies	Patients, N	Relative Risk (95% CI)	P	Heterogeneity(P; I^2^)
Baseline APACHE II score					
Both groups < 20	5	541	0.69 (0.45, 1.04)	0.08	0.45; 0%
Both groups ≥ 20	5	888	0.82 (0.66, 1.04)	0.10	0.76; 0%
One group ≥ 20	2	34	0.39 (0.15, 1.02)	0.06	0.57; 0%
SAPS II	2	657	0.92 (0.73, 1.16)	0.50	1.00; 0%
Baseline age (years)					
≤ 50	8	695	0.71 (0.48, 1.04)	0.08	0.69; 0%
> 50	7	1435	0.86 (0.73, 1.01)	0.06	0.45; 0%
Number of patients					
< 20/group	4	60	0.60 (0.21, 1.70)	0.34	0.36; 7.34%
≥ 20/group	11	2070	0.84 (0.72, 0.98)	0.03	0.72; 0%
Publication year					
Before 2010	8	465	0.71 (0.40, 1.27)	0.25	0.47; 0%
After 2010	7	1665	0.84 (0.72, 0.98)	0.03	0.61; 0%
Pathogen					
Mostly Gram-negative	11	1323	0.81 (0.68, 0.96)	0.02	0.68; 0%
Mix	4	807	0.87 (0.52, 1.45)	0.59	0.33; 12.27%
Loading dose in prolonged infusion group					
Yes	13	2020	0.84 (0.72, 0.97)	0.02	0.55; 0%
No	2	110	0.65 (0.24, 1.73)	0.39	0.54; 0%
Classification of antibiotics					
Carbapenems	4	907	0.90 (0.73, 1.10)	0.30	0.58, 0%
Cephalosporins	3	128	0.79 (0.22, 2.89)	0.73	0.14, 48.95%
Penicillin	5	463	0.61 (0.38, 0.98)	0.04	0.93, 0%

*APACHE II* Acute Physiology and Chronic Health Evaluation II, *SAPS II* Simplified Acute Physiology Score II

### Heterogeneity, publication bias, and sensitivity analysis

No statistically significant heterogeneity was found among the studies that evaluated mortality (I^2^ = 0%, P = 0.66), microbiological success (I^2^ = 22.2%, P = 0.27), or adverse events (I^2^ = 0%, P = 0.61). However, moderate heterogeneity was observed in the studies that evaluated clinical success (I^2^ = 52.4%, P = 0.03). No significant indication of publication bias was found for any outcome. This finding was supported by the results of the funnel plot and Egger’s test (P > 0.05) (Additional file 3: Figure S3). To assess the robustness of the results, a sensitivity analysis was conducted by omitting one study at a time and calculating the pooled effect sizes for the remaining studies. The direction and magnitude of the pooled estimates remained consistent with the omission of any single study, indicating that the meta-analysis was reliable and the results were robust (Additional file 4: Figure S4).

## Discussion

Numerous meta-analyses have investigated the clinical benefits of prolonged and intermittent infusion of β-lactam antibiotics; nevertheless, the outcomes were inconsistent, and no definitive conclusions were reached [9, 14, 35–38]. This meta-analysis provides an updated review of RCTs in patients with sepsis to determine whether prolonged infusion offers a clinical advantage in terms of mortality and clinical success. Our results demonstrate that, compared to intermittent administration, prolonged infusion leads to a 17% reduction in all-cause mortality and an improvement in clinical success without a notable increase in adverse events. Our findings align with the International consensus, which recommends prolonged infusion of β-lactam antibiotics in critically ill adults to reduce mortality and improve clinical cure rates, providing further support for these recommendations [15]. Microbiological eradication, compared to clinical success, objectively reflects the efficacy of β-lactam antibiotics. However, data regarding microbiological success were available from only five studies, and this limited number of included studies makes it challenging to draw meaningful conclusions. This could be attributed to the fact that, unlike mortality and clinical success, microbiological assessment requires support from bacteriological documentation. The limited number of patients with isolated causative microorganisms has resulted in a deficiency of statistical power in the majority of studies within the field of microbiology [15].

As a time-dependent antibiotic, the antibacterial effectiveness of β-lactam antibiotics is closely linked to the duration during which the drug concentration remains above the MIC. Optimal bactericidal activity was defined as the time during which the free drug concentration remained above the MIC for at least 40–70% of the total exposure time [39]. This aspect is especially crucial for critically ill patients with infections. According to International consensus recommendations, in order to better achieve microbiologic targets for bacterial killing, it is advised to maintain 100% fT > MIC and drug concentrations should exceed up to four to eight times free drug over the steady-state concentration (fC_ss_) when administering β-lactam antibiotics through continuous infusion [15]. Hence, some studies support the use of prolonged infusion (i.e., extended or continuous infusion) of β-lactam antibiotics to enhance treatment effectiveness and increase the chances of achieving maximum bactericidal activity [11], thereby improving patient outcomes.

To explore the impact of various factors on mortality, we conducted a multilevel subgroup analysis to gain a deeper understanding of the efficacy of β-lactam antibiotics in various patient populations. Inconsistent with the findings of Vardakas [9], our study shows that prolonged infusion of β-lactam antibiotics tends to reduce all-cause mortality compared to intermittent infusion, regardless of patient age being over 50, though without statistical significance. It is important to note that Vardakas’s study specifically focused on antipseudomonal β-lactams and not all included patients were diagnosed with sepsis. Concerning APACHE II scores, our results suggest a favorable trend in mortality reduction with prolonged infusion in critically ill patients, irrespective of an APACHE II score above or below 20, differing from Roberts’ meta-analysis findings [38]. In Roberts’ study, critically ill patients with an APACHE II score over 22 showed a trend of reduced hospital mortality with continuous infusion (RR, 0.74; 95% CI 0.53–1.01; P = 0.06). Yet, for those with a score below 22, continuous and intermittent infusion had comparable outcomes (P = 0.19). The 2023 International consensus [15] recommends preferring prolonged over short infusion of β-lactam antibiotics to lower mortality or enhance clinical cure, notably in critically ill adults. In studies involving non-critically ill patients, a systematic review shows that prolonged infusion does not improve all-cause mortality in febrile neutropenia patients [40]. Another meta-analysis of non-critically ill patients, encompassing 6 RCTs on mortality, found no survival difference between prolonged and short infusion (RR, 1.06; 95% CI 0.52–2.18; P = 0.61) [15]. Consequently, prolonged infusion of β-lactam antibiotics may reduce mortality and improve clinical cure rates in critically ill patients. However, its routine use is not advised for non-critically ill patients. Abdul-Aziz et al. emphasized in their review [41] that low methodological study quality and small sample sizes in RCTs could lead to heterogeneity between studies, resulting in non-significant results. In our analysis, all the included RCTs exhibit high methodological quality. The analysis includes 465 patients from 8 RCTs conducted before 2010 and 1,665 patients from 7 RCTs after 2010. Variations in subgroup analysis results likely stem from sample size differences. This highlights the need for future large-sample RCTs to comprehensively assess prolonged infusion of β-lactam antibiotics’ impact on the mortality of critically ill patients.

Moreover, our results indicate that patients with Gram-negative bacterial infections experience lower mortality rates when treated with prolonged infusions. The unique outer membrane in Gram-negative bacteria consist of lipopolysaccharides and a thinner peptidoglycan cell wall in the periplasmic space [42]. β-lactam antibiotics target the bacterial cell wall and, unlike drugs that act on the cytoplasm, more easily reach their targets [43]. The high accessibility of β-lactam antibiotics to the bacterial cell wall may be a significant contributing factor to the substantial reduction in mortality observed when treating patients with Gram-negative bacterial infections. When analyzing β-lactam subclasses, no significant differences in outcomes were observed, except for the penicillin subclass analysis, wherein reduced mortality in the prolonged infusion group was observed. Similar conclusions have been reported in another study [44] and in systematic reviews [9, 35]. However, owing to the limited number of studies included, additional data are needed to evaluate subclass variations.

Prolonged infusion of a loading dose is linked to improved clinical outcomes in critically ill patients with sepsis and septic shock [23, 28, 30]. Our results are consistent with prior meta-analyses [9, 36] and supported by the International consensus recommendations [15]. The consensus advocates for using a loading dose at the start of continuous infusion β-lactam antibiotics to enhance clinical success and lower mortality. In critically ill patients, particularly during the early phases of severe sepsis and septic shock, pathophysiological changes alter the pharmacokinetics of β-lactam antibiotics [45]. These patients experience an increased volume of distribution and accelerated drug clearance, resulting in lower initial drug concentrations [8, 45]. Consequently, administering a loading dose before the continuous infusion of β-lactam antibiotics can help maintain drug concentrations above the MIC and reduce the risk of treatment failure [11].

In addition to the existing studies, we compiled ongoing RCTs sourced from the International Clinical Trials Registry Platform (ICTRP) and ClinicalTrials (Table 3). Among these trials, the BLING III trial [46], which is near completion, is a phase 3 study involving 7203 critically ill patients with sepsis. This trial was designed to compare the impact of continuous and intermittent infusions of piperacillin-tazobactam or meropenem on the mortality status of patients with sepsis assessed 90 days after randomization. We anticipate that the forthcoming results from these large-scale clinical RCTs will provide a wealth of compelling evidence on whether prolonged infusion of β-lactam antibiotics improves mortality in septic patients.

**Table 3:** Ongoing trials of septic patients treated with prolonged and intermittent infusion of β-lactam antibiotics

Study	Date of first enrolment	Type	Country	Patients	β-Lactam antibiotics	Primary outcomes	ID of the trial	Sample, N	Phase
ANZCTR	2010/4/14	RCT	Australia, Hongkong	Severe sepsis	Ticarcillin/clavulanate, Piperacillin/tazobactam, Meropenem	Plasma antibiotic concentration above the MIC for three samples taken on days 3 and 4	ACTRN12610000238077	60	2
PACTR	2020/9/21	RCT	South Africa	Adult and pediatric with Sepsis	Amoxicillin/clavulanate, Piperacillin/tazobactam, Meropenem, Imipenem	Pediatrics: the proportion of patients in the IB group with an expected time above MIC. Adults: clinical cure rates at day 14	PACTR202009811610400	408	NA
BLING III	2018/3/26	Open-label RCT	Australia, New Zealand, the United Kingdom, Belgium	Sepsis	Piperacillin/tazobactam, Meropenem	Mortality at day 90	NCT03213990	7203	3
BICCS	2023/8/1	Open-label RCT	France	Sepsis or septic shock	β-Lactam antibiotics	Mortality at day 30	NCT05681442	600	4
PROBES	2021/9/20	Open-label RCT	China	Early Septic Patients	β-Lactam antibiotics	All-cause mortality in ICU; 28-day all-cause mortality	NCT05024565	2600	NA
PAACS	2018/7/16	Open-label; Non-RCT	France	Early Septic Patients	Piperacillin/tazobactam, Cefepime, Meropenem	Proportion of patients achieving the PK/PD target; all-cause mortality	NCT02820987	129	3

*MIC* minimum inhibitory concentration, *IB* intermittent beta-lactam dosing, *RCT* Randomized Controlled Trials, *PK/PD* Pharmacokinetics/Pharmacodynamics, *NA* Not Applicable

Our systematic review has several strengths compared to previous studies. First, we applied a strict definition of sepsis. The included studies adhered to the sepsis criteria at the time of the study. Secondly, to investigate the impact of various factors on mortality, we conducted a multilayered subgroup analysis. This approach is valuable for tailoring distinct clinical treatment strategies for different subgroups of patients with sepsis. Third, we incorporated the latest results from two large-scale RCTs [12, 13], such as the MERCY trial [13], which is the largest RCT to date on this topic, including 607 patients with sepsis. By combining the most recent data, we aimed to provide a comprehensive and systematic evaluation of the efficacy and safety of β-lactam antibiotics in sepsis.

However, our meta-analysis has several limitations. First, outcome definitions, such as mortality and clinical success, differed among studies, possibly contributing to heterogeneity in results. Furthermore, critically ill patients presented with complex conditions and multiple comorbidities that could introduce individual variations that might have affected the outcomes of the study. Additionally, the spanning of included studies across a wide range of years could introduce clinical heterogeneity due to evolving sepsis definitions, possibly affecting result consistency.

## Conclusion

Compared to intermittent infusion, prolonged infusion of β-lactam antibiotics significantly decreases all-cause mortality among patients with sepsis and enhances clinical success without increasing adverse events. The conclusions of our meta-analysis are in line with the International consensus recommendations. This alignment not only signifies the reliability of our research methods and analysis but also offers strong scientific backing for the recommendations.

### Supplementary Information

**Additional file 1.** Risk of bias graph.

**Additional file 2.** Risk of bias summary.

**Additional file 3.** Funnel plot.

**Additional file 4.** Sensitivity analysis.

## Data Availability

Not applicable.

## Abbreviations

RCT: Randomized controlled trial
RR: Relative risk
CI: Confidence interval
APACHE II: Acute Physiology and Chronic Health Evaluation II
SAPS II: Simplified Acute Physiology Score II
MIC: Minimum inhibitory concentration
PRISMA: Preferred Reporting Items for Systematic Reviews and Meta-Analysis
PI: Prolonged infusion
II: Intermittent infusion
PK/PD: Pharmacokinetics/Pharmacodynamics
